# Emergency department and inpatient health care utilization among patients who require interpreter services

**DOI:** 10.1186/s12913-015-0874-4

**Published:** 2015-05-29

**Authors:** Jane W. Njeru, Jennifer L. St. Sauver, Debra J. Jacobson, Jon O. Ebbert, Paul Y. Takahashi, Chun Fan, Mark L. Wieland

**Affiliations:** Division of Primary Care Internal Medicine, Mayo Clinic, 200 First Street, SW, Rochester, MN 55905 USA; Division of Epidemiology, Mayo Clinic, Rochester, MN USA; Division of Biomedical Statistics and Informatics, Mayo Clinic, Rochester, MN USA; Mayo Clinic Robert D. and Patricia E. Kern Center for the Science of Health Care Delivery, Rochester, MN USA

**Keywords:** Health disparities, Health services research, Limited English proficiency

## Abstract

**Background:**

Limited English proficiency is associated with health disparities and suboptimal health outcomes. Although Limited English proficiency is a barrier to effective health care, its association with inpatient health care utilization is unclear. The aim of this study was to examine the association between patients with limited English proficiency, and emergency department visits and hospital admissions.

**Methods:**

We compared emergency department visits and hospitalizations in 2012 between patients requiring interpreter services and age-matched English-proficient patients (who did not require interpreters), in a retrospective cohort study of adult patients actively empanelled to a large primary health care network in a medium-sized United States city (n = 3,784).

**Results:**

Patients who required interpreter services had significantly more Emergency Department visits (841 vs 620; *P* ≤ .001) and hospitalizations (408 vs 343; *P* ≤ .001) than patients who did not require interpreter services. On regression analysis the risk of a first Emergency Department visit was 60 % higher for patients requiring interpreter services than those who did not (unadjusted hazard ratio [HR], 1.6; 95 % confidence interval (CI), 1.4-1.9; *P* < .05), while that of a first hospitalization was 50 % higher (unadjusted HR, 1.5; 95 % CI, 1.2-1.8; *P* < .05). These findings remained significant after adjusting for age, sex, medical complexity, residency and outpatient health care utilization.

**Conclusions:**

Patients who required interpreter services had higher rates of inpatient health care utilization compared with patients who did not require an interpreter. Further research is required to understand factors associated with this utilization and to develop sociolinguistically tailored interventions to facilitate appropriate health care provision for this population.

## Background

In 2010, approximately 9 % of the United States population had limited English proficiency (LEP), which is defined by the US Census Bureau as speaking English “less than very well” in any person 5 years of age or older [1]. The number of immigrants with LEP increased by 80 % in the past 2 decades, and this trend is projected to continue [2]. Persons with LEP require medical interpreters or language-concordant providers and staff for effective and efficient interactions with health care systems [3].

Limited English proficiency is an important mediator of health disparities, and has been linked to overall poor health and low quality of health care delivery [4]. It is associated with limited access to health care [5, 6], decreased understanding of medical information [7], and lower use of preventive services [5, 8]. Compared with patients who speak English well, LEP is associated with suboptimal disease-specific outcomes in mental health [9], asthma [10], diabetes mellitus [11], and heart failure [12]. Further, LEP has been linked to patient dissatisfaction with care received [13]. These disparities are only partially mitigated by appropriate use of medical interpreters [14] or provision of 100 % language-concordant providers [11]. Patient-centered medical homes in primary care practices across the country aim to promote more effective health care utilization while improving care through integrated systems [15, 16]. Among patients with LEP, efficient and effective health care utilization may be challenging, and patient-centered medical homes have the potential to reduce health inequity through care coordination and other mechanisms [15].

Although LEP is a barrier to optimal health care access among adults with access to the health care system, those who require medical interpreters have more primary care outpatient visits than those who do not [17]. Emergency department (ED) visits and hospitalizations account for a significant proportion of total health care costs in the United States. Available data on health care utilization among persons with LEP is limited to data focusing on specific medical conditions such as asthma [10] and congestive heart failure [18] in elderly patients, readmission risks among general medical in-patients, [19] and specific utilizations events such as length of hospital stay and home health referrals following dismissal [20–22]. The results of these studies indicate possible higher and less efficient healthcare utilization among patients with LEP compared with patients who speak English well. Use of language congruent providers and interpreters has been shown to result in less cost for ED visits among children [23], while improved access and utilization of primary care services has been associated with less ED visits [5, 24, 25]. More data on overall inpatient health care utilization among patients with LEP are needed to help shape interventions within primary care practices and medical homes to improve health care utilization among these patients.

To advance our understanding of health care utilization among patients with LEP, we conducted a population-based study of ED visits and hospitalizations among patients with LEP in a primary care setting. We hypothesized that patients who require interpreters would have higher utilization of ED visits and hospitalization.

## Methods

This study was approved by the Institutional Review Boards at Mayo Clinic and Olmsted Medical Center. All participants were adults, who gave general written authorization for the use of their medical records for research purposes, per the Minnesota state privacy law, Statute 144.335 [26].

### Study population

We conducted a retrospective cohort study of patients empanelled in the Primary Care Internal Medicine and Family Medicine practices at Mayo Clinic (Rochester, Minnesota). These practices provide primary care in a patient-centered medical home to approximately 135,000 patients in Olmsted County, Minnesota. Olmsted County has a population of 147,161 (2012 estimate), with the following distribution: White: 82.5 %, Asian: 5.8 %, Black or African American: 5.3 %, American Indian or Alaska Native: 0.3 %, Native Hawaiian or Pacific Islander: 0.1 % and Hispanic or Latino: 4.4 %. In 2012, 12.6 % of the population spoke a language other than English at home [27]. To be eligible for the study, patients had to be empanelled to this practice and be seen by a health care provider at least once from January 1 through December 31, 2012. Patients had to be at least 18 years old at the beginning of the study period.

From the pool of eligible patients, we identified those who required interpreter services (IS) and the language spoken by using an institutional administrative database and the electronic medical record. Patients self-identified the need for IS. Mayo Clinic provides trained medical interpreters in person or by phone to facilitate communication between 2 parties by interpreting language and culture and by conveying the message accurately without adding, modifying, or deleting information. The cohort of non-IS patients (patients whose electronic medical record did not contain the IS flag) were age-frequency-matched to the IS cohort.

### Data collection

Olmsted County, Minnesota, has 3 hospitals, 2 with EDs; all are geographically isolated from the next-closest inpatient facilities. The majority of inpatient utilization among patients in our study sample was expected to occur at 1 of these 3 facilities. Using the Rochester Epidemiology Project medical records linkage system [28], we electronically identified hospital admissions and ED visits using billing records. Data was obtained from patient registration information, billing records, and chart reviews.

### Measures

Independent variables:

1. Demographic information: age, sex, marital status, ethnicity, race
2. Language spoken and Interpreter Status: The language spoken by patients was identified from registration information. Patients who require interpreter services have an easily identifiable flag in the medical record, which also identifies their language
3. Insurance type (government or non-government)
4. Residency (rural or urban)
5. Patient medical complexity. This was measured with the Charlson comorbidity index [29], which considers the number and severity of 19 predefined comorbid conditions (as identified by ICD-9 codes) and provides a weighted score of a patient’s comorbidities. This score can be used to predict short- and long-term outcomes such as function, length of hospitalization, and mortality rates [30]. We used the time period of 5 years before baseline to identify diagnoses used to calculate this score.

Dependent variables:

6. Number of ED visits and hospital admissions during the 12-month study interval
7. Outpatient utilization (number of outpatient visits to Primary Care Internal Medicine or Family Medicine during the 12-month study period)

A manual chart review was performed by 1 author (J.W.N.) to confirm each ED visit and hospitalization and to record the primary reason (diagnosis) for each utilization event. These diagnoses were then clustered into pre-determined system-based diagnostic categories for analysis. Using International Classification of Diseases, Ninth Revision (ICD-9) codes, pregnancy-related utilization events were excluded from the analysis.

### Statistical methods

Descriptive analyses were used to present demographic characteristics by IS status, using estimates of frequencies for categorical variables and medians and interquartile range for continuous variables. These were compared by IS status using a χ^2^ test for categorical variables and *t* tests or rank sum tests for continuous variables. For descriptive purposes, the number of out-patient, ED and hospitalization visits was categorized and compared using a χ^2^ test. Proportional hazard regression was used to assess the association between IS status and first ED visit or hospitalization, and results were presented as hazard ratios (HRs) with 95 % Confidence Intervals (CI). Multivariable models were used to adjust for the effect of age, sex, marital status, Charlson comorbidity index, number of outpatient visits and residency. Additional stratified models were used to assess for differences in the association among sub-groups. Variables assessed included age quartiles, sex, residence (urban vs. rural), Charlson comorbidity index (0, 1, and 2+) and number of outpatient visits (0–1, 2–4, 5–10, and 11+). Stratified models are presented only for those where a possible interaction was observed. Missing data were minimal. The primary diagnosis for each ED visit and hospitalization were sorted according to body system and compared between IS and non-IS patients using a χ^2^ test. All analyses were performed using SAS version 9.2 (SAS Institute Inc., Cary, NC). *P* values of .05 or less were considered statistically significant.

## Results

We identified 88,768 eligible adult patients. Of these, 207 (0.2 %) did not grant permission for their records to be used for research and were excluded. Of the remaining 88,561 patients, 1,892 (2.1 %) had used IS. An age-frequency-matched group of non-IS patients was identified, for a total study group of 3,784 patients. Patient characteristics are shown in Table 1. In contrast to the IS group, non-IS patients were more likely to be of white race and of non-Hispanic or Latino ethnicity. A total of 40 different languages were represented in the IS group, and the 5 most common languages represented approximately two-thirds of the entire IS population (Somali 30.7 %; Spanish 15.2 %; Vietnamese 11.7 %; Khmer 10.1 % and Arabic 9.3 %).

**Table 1:** Patient characteristics (N = 3,784)

Characteristic	IS Patients (n = 1,892)	Non-IS Patients (n = 1,892)	*P* Value^a^
Age, median (IQR), y	51.0 (36.0-64.0)	51.0 (35.0-64.0)	.95
Male sex, No. of patients (%)	718 (37.9)	821 (43.4)	<.001
Race, No. of patients (%)			<.001
American Indian or Alaska Native	12 (0.6)	4 (0.2)	
Black	511 (27.0)	34 (1.8)	
White	235 (12.4)	1,726 (91.2)	
Native Hawaiian or Pacific Islander	9 (0.5)	1 (0.1)	
Asian	511 (27.0)	50 (2.6)	
Other, unknown, or chose not to disclose	614 (32.5)	77 (4.1)	
Ethnicity, No. of patients (%)			<.001
Hispanic or Latino	259 (13.7)	29 (1.5)	
Not Hispanic or Latino	1,325 (70.0)	1,698 (89.7)	
Unknown or chose not to disclose	308 (16.3)	165 (8.7)	
Marital status, No. of patients (%)			<.001
Married or life partner	1,164 (61.5)	1,267 (67.0)	
Divorced, single, widowed, separated, or unknown	728 (38.5)	625 (33.0)	
Insurance			<.001
Government	1,024 (54.1)	550 (29.1)	
Non-Government	505 (26.7)	1,160 (61.3)	
Unknown	363 (19.2)	182 (9.6)	
Residency			<.001
Rural	132 (7.0)	686 (36.3)	
Urban	1,397 (73.8)	1,025 (54.1)	
Unknown	363 (19.2)	181 (9.6)	
Charlson Score^b^	N = 1860	N = 1857	0.0009
0	1127 (60.6)	1235 (66.5)	
1	366 (19.9)	307 (16.5)	
2+	367 (19.7)	315 (17.0)	
Out-patient visits			<0.0001
0–1	660 (34.9)	415 (21.9)	
2–4	357 (18.9)	515 (27.2)	
5–10	435 (23.0)	483 (25.5)	
11+	440 (23.3)	479 (25.3)	
Emergency department visits, No. of patients (%)			<.001
0	1,607 (84.9)	1,691 (89.4)	
1	181 (9.6)	141 (7.5)	
2	45 (2.4)	31 (1.6)	
≥3	59 (3.1)	29 (1.5)	
Hospitalizations, No. of patients (%)			<.001
0	1,443 (76.3)	1,600 (84.6)	
1	300 (15.9)	220 (11.6)	
2	80 (4.2)	42 (2.2)	
≥3	69 (3.6)	30 (1.6)	

*IQR* interquartile range, *IS* interpreter services

^a^χ^2^ Test for categorical variables and rank sum test for continuous variables

^b^Available for a subset of the population (*IS* patients, n = 1,860; non-*IS* patients, n = 1,857)

We observed significantly more total ED visits (841 vs 620; *P* ≤ .001) and hospitalizations (408 vs 343; *P* ≤ .001) for IS patients compared with non-IS patients during the study interval. Likewise, the proportion of patients with at least 1 ED visit (23.7 % vs 15.4 %; *P* ≤ .001) and at least 1 hospitalization (15.1 % vs 10.6 %; *P* ≤ .001) was significantly higher among IS patients (Table 1). Additionally, almost twice as many IS patients had 3 or more ED visits and hospitalizations than non-IS patients (Table 1).

IS patients had a 60 % higher risk of at least 1 ED visit (unadjusted HR, 1.6; 95 % CI, 1.4-1.9; *P* < .05) and a 50 % higher risk of at least 1 hospitalization (unadjusted HR, 1.5; 95 % CI, 1.2-1.8; *P* < .05). After adjusting for age, sex, marital status, Charlson comorbidity index, number of outpatient visits, and residency, these findings remained significant for the risk of a first ED visit (adjusted HR, 1.5; 95 % CI, 1.3-1.8; P < .05), and a first hospitalization (adjusted HR, 1.3; 95 % CI, 1.1-1.7; P < .05). We also performed a stratified analysis and found evidence of differences in risk of ED visits or hospitalizations due to age, sex, and residency (Table 2). Specifically, IS patients aged 18 to 36 years, were 3.7 times more likely to be hospitalized compared with non-IS patients (*P* < .0001) and female IS patients had an increased risk of ED visits (*P* = .03). IS residents from rural areas had an increased risk of hospitalizations (*P* = .02) (Table 2).

**Table 2:** Risk of ED visit or hospitalization for IS patients vs non-IS patients, stratified by age quartiles, sex and residency

	At Least 1 ED Visit		At Least 1 Hospitalization	
	Unadjusted HR (95 % CI)	Adjusted HR (95 % CI)^a^	Unadjusted HR (95 % CI)	Adjusted HR (95 % CI)^a^
Age Quartile^b^				
18-36 y	1.6 (1.2-2.1)	1.5 (1.1-2.0)	3.3 (2.1-5.1)	3.7 (2.2-6.1)
>36-51 y	1.6 (1.2-2.2)	1.5 (1.1-2.1)	1.0 (0.7-1.4)	0.6 (0.4-0.9)
>51-64 y	2.1 (1.5-2.8)	2.2 (1.5-3.1)	1.5 (1.0-2.1)	1.3 (0.8-2.0)
>64 y	1.3 (1.0-1.8)	1.3 (0.9-1.8)	1.2 (0.9-1.6)	1.7 (1.2-2.4)
All patients	1.6 (1.4-1.9)	1.5 (1.3-1.8)	1.5 (1.2-1.8)	1.3 (1.1-1.7)
Sex^c^				
Male	1.5 (1.2,1.9)	1.2 (0.9,1.6)	1.5 (1.1,2.1)	1.2 (0.9,1.7)
Female	1.9 (1.6,2.3)	1.7 (1.4,2.1)	1.5 (1.1,1.9)	1.5 (1.1,2.0)
Residency^d^				
Rural	1.1 (0.7,1.8)	0.9 (0.6,1.6)	2.7 (1.8,4.1)	2.3 (1.4,3.8)
Urban	1.7 .(1.4,2.0)	1.6 (1.4,1.9)	1.3 (1.1,1.6)	1.2 (1.0,1.5)

*ED* emergency department, *HR*, hazard ratio, *IS* interpreter services

^a^Adjusted for age, sex, marital status, Charlson comorbidity index, number of outpatient visits, and res residency

^b^Interaction p-value <0.0001 for hospitalization and 0.2741 by ED visits (adjusted model)

^c^Interaction p-value = 0.3439 for hospitalization and 0.0287 by ED visits (adjusted model)

^d^Interaction p-value = 0.0185 for hospitalization and 0.0542 by ED visits, (adjusted model)

Finally, we found that the reasons for the first hospitalization differed between IS patients and non-IS patients (Table 3). The three most common reasons for ED visits in both groups were musculoskeletal, infections, and gastrointestinal related diagnoses. This remained similar for the first hospitalization for the IS group. However, in the non-IS group, hospitalizations for infection related diagnoses was less common, while those for musculoskeletal diagnoses were more common among non-IS patients. The most common musculoskeletal diagnoses among the Non-IS patients, which explained the difference noted between the two groups, was elective joint replacements, mainly of the knee and hip.

**Table 3:** Indications for first ED visit and hospitalization

Visit Indication	IS Patients No. (%)	Non-IS Patients No. (%)	*P* value
ED	n = 449	n = 292	.2043
Musculoskeletal	98 (21.8)	63 (21.6)	
Infection	77 (17.1)	44 (15.1)	
Gastrointestinal	71 (15.8)	34 (11.6)	
Neurologic	39 (8.7)	17 (5.8)	
Trauma, assault	38 (8.5)	26 (8.9)	
Opthalmologic, dental, dermatology, ENT	34 (7.6)	22 (7.5)	
Cardiovascular	25 (5.6)	21 (7.2)	
Respiratory	20 (4.5)	14 (4.8)	
Renal, genitourinary, urinary	19 (4.2)	17 (5.8)	
Psychiatric, substance abuse, drug overdose	10 (2.2)	13 (4.5)	
Hematologic, PE, DVT	3 (0.7)	3 (1.0)	
Study, drug reactions, allergy, other	5 (1.1)	4 (1.4)	
Functional symptoms, falls	4 (0.9)	11 (3.8)	
Diabetes mellitus, endocrine	6 (1.3)	3 (1.0)	
Hospitalization	n = 285	n = 201	.0151
Infection	51 (17.9)	19 (9.5)	
Musculoskeletal^a^	45 (15.8)	48 (23.9)	
Gastrointestinal	40 (14.0)	25 (12.4)	
Cardiovascular	28 (9.8)	30 (14.9)	
Neurologic	25 (8.8)	15 (7.5)	
Psychiatric, substance abuse, drug overdose	17 (6.0)	18 (9.0)	
Renal, genitourinary, urinary	17 (6.0)	6 (3.0)	
Trauma, assault	16 (5.6)	6 (3.0)	
Opthalmologic, dental, dermatologic, ENT	16 (5.6)	6 (3.0)	
Hematologic, PE, DVT	11 (3.9)	10 (5.0)	
Respiratory	10 (3.5)	7 (3.5)	
Study, drug reactions, allergy, other	4 (1.4)	6 (3.0)	
Diabetes mellitus, endocrine	3 (1.1)	2 (1.0)	

*DVT* deep venous thrombosis, *ED* emergency department, *ENT*, ear-nose-throat, *IS* interpreter services; *PE* pulmonary embolism

^a^includes elective arthroplasties (*IS* n = 6; non-*IS* n = 18)

## Discussion

We observed that patients requiring IS had significantly higher patterns of inpatient utilization (ED visits and hospitalizations) compared with patients who did not require IS. Our findings are consistent with those of previous studies that documented higher inpatient utilization among patients with LEP for psychiatric disorders [22], coronary artery disease, some surgical syndromes [21], and pediatric ED visits [31].

Persons with LEP are heterogeneous with regards to culture, ethnicity, race, and sociodemographic factors [2]. Therefore, the reasons underlying increased inpatient utilization are likely multifaceted. Furthermore, this study cannot fully assess whether the excess utilization is “too much” care or the correct amount of care. Nevertheless, this study provides important objective findings of utilization in the context of existing literature around determinants of inpatient and emergency room utilization among patients with LEP.

Because most patients in our study had health insurance, were empanelled to a primary care practice, and were regular utilizers of the outpatient practice, some of the traditional barriers to health care access and availability cannot explain our findings. Organizational solutions to promote more efficient health care utilization must consider patient factors that frequently coexist with LEP, including low socioeconomic position, preexisting health care norms, and low health literacy [32–34].

Healthcare-seeking behaviors among patients with LEP may be influenced by the norms of their countries of origin. For example, the notion of chronic disease management and preventive care may be unfamiliar to some patients coming from a region where healthcare is defined as an acute care model [35, 36]. This may be associated with delays in seeking care, and as noted in one pediatric ED, LEP patients were more likely to be triaged to higher acuity, which led to hospitalization [37]. Our study lacks data on immigration status, which has been postulated to impact utilization of healthcare services, leading to delay in seeking care and recourse to ED visits [38]. However, other work suggests that undocumented immigrants in the US have similar levels of ED use to other immigrant and non-immigrant groups [39].

Finally, LEP and low health literacy are interrelated and often occur together [40]. LEP is associated with lower health literacy across different diseases, ethnicities, and ages [41]. Health literacy, in turn, has been independently linked to health care utilization [42, 43], and lower health literacy is associated with an inefficient mix of services, leading to higher health care costs [44]. Nevertheless, one study of 48,000 patients showed LEP to be an even more important risk factor for poor health than low health literacy [40]. Thus, in addition to language considerations, interventions aimed at improving the efficiency of health care utilization among patients with LEP should also incorporate principles of messaging and communication for patients with low health literacy [37, 45, 46].

Communication barriers and unmet health care needs may help explain the increased ED visits among patients with LEP [7]. ED visits among patients empaneled to a primary care practice are frequently preceded by communication between the patient and the outpatient health care team. Patients with LEP may be less likely to initiate this communication (typically a telephone call) if IS are less consistent. Indeed, language barriers during emergency telephone communications can negatively affect communication and care outcomes [47]. Likewise, provision of language-concordant outpatient providers for patients with diabetes mellitus in one study resulted in reduced ED visits and hospitalizations [48].

ED utilization may represent unmet health care needs among patients withLEP. Primary care practices are increasingly developing medical home initiatives for care coordination, integrated behavioral health, and care management for patients with complex medical problems [39, 49]. However, if special efforts are not taken, these programs may inadvertently exclude patients with LEP, who have difficulty navigating these complex systems-within-systems [50]. Therefore, ED visits may be a mechanism by which patients with LEP disproportionately address these unmet health care needs [51, 52]. This conclusion is further supported by our finding of higher ED utilization among IS patients for dental, eye, skin, and ENT concerns, systems that are typically addressed in the outpatient setting. Primary care practices should aim to systematically identify and manage patients with LEP who frequently utilize the ED and hospital, while improving communication to patients at these critical transitions that are linguistically, culturally, and health literacy–level appropriate.

Our finding of increased hospitalizations among IS patients is compelling in that the decision to be hospitalized is influenced largely by diagnostic circumstance and the decision making of the admitting physician, rather than the decision making of the patient. Communication between patients and providers is a key factor in the evaluation of patients at the point of care. Compared with English-proficient patients, more tests are ordered for patients with LEP who present to the ED with abdominal pain [53] and acute respiratory illnesses, and patients with LEP are more likely to receive antibiotics than non-LEP patients [54]. The decision to order extra tests or more aggressive therapy by ED providers may be influenced by a need to compensate for communication barriers, and this approach may then extend to the decision about whether a patient should be hospitalized. One study of pediatric patients showed higher admission rates among patients with LEP compared with English-proficient patients, even where acuity was similar at presentation [55].

Our study has several limitations. First, it was retrospective and relied on medical records. However, we had minimal missing data, and charts with any ED visit and hospitalization were reviewed to confirm the event. It is conceivable that ED visits and hospitalizations outside the 3 main local hospitals would be missed; however, we suspect such events would be minimal among these community-dwelling primary care patients. Limitations of our administrative dataset precluded the assessment of potentially important confounding variables such as socioeconomic position and health literacy. The use of IS need as a proxy for LEP is incomplete and represents only a subset of patients who truly have LEP [49]. Furthermore, the fact that IS status was assessed by self-report may have led to misclassification of patients. In addition, we are not able to verify the percentage of eligible patients who received IS services during health care events, though institutional policy dictates that professional interpreters participate in every clinical encounter. Also, insurance status, language and race/ethnicity are highly correlated and LEP is a marker for these characteristics as well; therefore, it is not possible to separate out the individual effects of these factors in our study. While we did calculate the Charlton Comorbidity Index for each patient and incorporated it into our analyses, we do not have access to data regarding the acuity of conditions that prompted each utilization event. Finally, this study was conducted among patients and institutions in a single geographic region within a medical home, with implications for generalizability to other primary care practices. Likewise, these results may not be applicable to practices with much higher percentages of IS patients that may have systems in place that specifically target inpatient utilization among patients with LEP.

## Conclusion

In summary, among patients empanelled to a large primary care practice, patients who required IS had significantly higher utilization of inpatient health care services (ED visits and hospitalizations) compared with those who did not need IS. Additional research is needed within primary care practices and medical homes to implement socio-linguistically tailored interventions that improve the efficiency of health care utilization among patients with LEP while acknowledging the heterogeneity of attitudes and behaviors among these populations that may vary according to multiple factors, including gender, ethnicity, and duration of residence in the U.S. By addressing the reasons for utilization inefficiency, interventions should aim to reduce health disparities among patients with LEP while reducing utilization events and health care cost.

## Abbreviations

CI: Confidence interval
ED: Emergency department
ENT: Ear-nose-throat
HR: Hazard ratio
ICD-9: International classification of diseases, ninth revision
IS: Interpreter services
LEP: Limited English proficiency
